# Role of Fractalkine/CX3CR1 Interaction in Light-Induced Photoreceptor Degeneration through Regulating Retinal Microglial Activation and Migration

**DOI:** 10.1371/journal.pone.0035446

**Published:** 2012-04-19

**Authors:** Meng Zhang, Gezhi Xu, Wei Liu, Yingqin Ni, Wenting Zhou

**Affiliations:** 1 Department of Ophthalmology, Eye and ENT Hospital of Fudan University, Shanghai, People's Republic of China; 2 Institute of Brain Science, Fudan University, Shanghai, People's Republic of China; 3 State Key Laboratory of Medical Neurobiology, Fudan University, Shanghai, People's Republic of China; Massachusetts Eye & Ear Infirmary, Harvard Medical School, United States of America

## Abstract

**Background:**

Excessive exposure to light enhances the progression and severity of some human retinal degenerative diseases. While retinal microglia are likely to be important in neuron damage associated with these diseases, the relationship between photoreceptor damage and microglial activation remains poorly understood. Some recent studies have indicated that the chemokine fractalkine is involved in the pathogenesis of many neurodegenerative diseases. The present study was performed to investigate the cross-talk between injured photoreceptors and activated retinal microglia, focusing on the role of fractalkine and its receptor CX3CR1 in light-induced photoreceptor degeneration.

**Methodology/Principal Findings:**

Both in vivo and in vitro experiments were involved in the research. In vivo, Sprague–Dawley rats were exposed to blue light for 24 hours. In vitro, the co-culture of primary retinal microglia and a photoreceptor cell line (661W cell) was exposed to blue light for five hours. Some cultures were pretreated by the addition of anti-CX3CR1 neutralizing antibody or recombinant fractalkine. Expression of fractalkine/CX3CR1 and inflammatory cytokines was detected by immunofluorescence, real-time PCR, Western immunoblot analysis, and ELISA assay. TUNEL method was used to detect cell apoptosis. In addition, chemotaxis assay was performed to evaluate the impact of soluble fractalkine on microglial migration. Our results showed that the expression of fractalkine that was significantly upregulated after exposure to light, located mainly at the photoreceptors. The extent of photoreceptor degeneration and microglial migration paralleled the increased level of fractalkine/CX3CR1. Compared with the control, the expression of inflammatory cytokines was significantly downregulated in the anti-CX3CR1 neutralizing antibody-treated group, and the number of photoreceptors was also well preserved. The addition of recombinant full-length fractalkine or soluble fractalkine resulted in fewer TUNEL-positive photoreceptors and an increased number of migratory microglia respectively.

**Conclusions/Significance:**

These findings demonstrate that fractalkine/CX3CR1 interaction may play an important role in the photoreceptor-microglia cross-talk in light-induced photoreceptor degeneration.

## Introduction

Excessive exposure to light enhances the progression and severity of some human retinal degenerative diseases, such as age-related macular degeneration, and some forms of retinitis pigmentosa due to irreversible photoreceptor apoptosis [1]. Understanding the process of photoreceptor apoptosis might provide evidence for how to interfere with photoreceptor loss and therefore loss of vision in these diseases.

Microglia located in the inner retina are defined as the resident macrophages of the central nervous system (CNS) [2]. Some evidence indicates that microglial activation contributes to neuron damage in neurodegenerative diseases [3]–[5]. In response to certain environmental changes, microglia can become overactivated and therefore exert detrimental neurotoxic effects by the excess production of cytotoxic factors [6]–[8]. Our previous finding suggests that inhibition of microglial activation by naloxone is neuroprotective in a model of light-induced photoreceptor degeneration [9]. Although the involvement of microglia is certain, the relationship between photoreceptor damage and microglial activation remains poorly understood [10].

Recently, an increasing number of studies have focused on the signal cross-talk between neurons and microglia [11]. Chemokines are a family of relatively low molecular mass proteins that chemo-attract and activate inflammatory cells [12]. Different from most other chemokines, fractalkine is a peculiar chemokine belonging to the CX3C subfamily that exists as both soluble and membrane-bound forms [13]. It is constitutively expressed by neurons throughout the CNS, whereas its sole receptor, CX3CR1, is restricted to major microglia in the CNS [14]–[16]. Under stimulation, the membrane-bound form of fractalkine on neurons can be cleaved into the soluble form by the activation of metalloproteases [17]–[18]. This pattern of expression makes the ligand–receptor pair fractalkine/CX3CR1 appear to be an ideal candidate to mediate neural/microglial interaction [19].

Recent studies show that fractalkine is involved in the pathogenesis of many clinical diseases including pancreatitis [20], rheumatoid arthritis [21], AIDS [22], atherosclerosis [23], [24], age-related macular degeneration [25]–[27], neuropathic pain [28]–[30], and cancer [31]–[33]. It is also suggested that the fractalkine/CX3CR1 in the CNS not only modulates the recruitment/activation of immune cells [34], [35], but might also exert multiple effects on neurons [36]–[38]. Indeed, the role of fractalkine/CX3CR1 is still quite controversial. For example, CX3CR1-deficient mice showed reduced inflammation and injury in models of cerebral ischemia [39]–[41], Alzheimer's disease (AD) [42], and mutant tau/APP-associated neurodegeneration [43]. On the other hand, loss of CX3CR1 exaggerates neuron apoptosis in Parkinson's disease [27], amyotrophic lateral sclerosis [27], and encephalomyelitis [44]. This indicates that fractalkine/CX3CR1 might play different and even opposite roles in different disease models [36].

In contrast to a tremendous amount of research on the brain, little is known about the role of fractalkine/CX3CR1 in light-induced photoreceptor degeneration. We hypothesized that photoreceptor damage due to excessive light exposure would result in membrane-bound form fractalkine cleavage into soluble form and then enhanced microglial activation, which would be driven by the further loss of photoreceptors throughout the degenerative process.

Therefore, the purpose of this study was to determine the role of fractalkine/CX3CR1 interaction in light-induced photoreceptor apoptosis. In this work, we investigated the changes in fractalkine/CX3CR1 expression after light damage, as well as the regulation of microglial functions mediated by the fractalkine/CX3CR1 ligand/receptor pair, in both the in vivo type I retinal photic injury model and the in vitro photoreceptor/microglia co-culture system. Our data show that the alteration in the forms of fractalkine protein expressed due to light damage exacerbates retinal inflammation and photoreceptor apoptosis by increased recruitment of activated microglia and excess production of inflammatory cytokines. In addition, the blockade of soluble-form fractalkine activity by means of a neutralizing monoclonal antibody partly delayed the degenerative process of photoreceptors and, in contrast, exogenous membrane-bound form fractalkine administration promoted photoreceptor survival, suggesting that the equilibrium between membrane-bound and soluble-form fractalkine plays an important role in the biological activity of fractalkine/CX3CR1. Taken together, these findings suggest that fractalkine/CX3CR1 represents a potential novel therapeutic target to regulate inflammation and photoreceptor survival in light-induced retinal degeneration.

## Materials and Methods

### Animals

All procedures were conducted in accordance with the ARVO statement for the Use of Animals in Ophthalmic and Vision Research, and the study was approved by the Animal Ethics Committee of the Eye and ENT Hospital of Fudan University. Adult male Sprague–Dawley rats, each weighing 200 to 230 g, were maintained in a 12-hour light/12-hour dark cycle. They were fed ad libitum and had free access to water.

### Cell Culture

#### Primary retinal microglia culture

Retinas were isolated from newborn Sprague-Dawley rats by mechanical dissociation and then incubated in 0.25% trypsin, 1 mM EDTA (Invitrogen, Carlsbad, CA) to generate single cell suspension. The enzyme-treated tissues were then triturated with a pipette and the trypsin was inactivated by fetal bovine serum (FBS) (Gibco, Carlsbad, CA). Cells were collected by centrifugation and re-suspended in DMEM/F-12 medium (Gibco, Carlsbad, CA) containing 10% FBS and 1% penicillin/streptomycin (Sigma-Aldrich, St. Louis, MO). The cells were seeded in 75 cm^2^ culture flasks and incubated at 37°C in a humidified 5% CO_2_ atmosphere. After two or three weeks of growth, mixed glial cells were shaken at 250 rpm for 4 hours and the supernatant containing an enriched microglia were harvested to reseed.

#### 661W cell culture

The 661W photoreceptor cell line was kindly provided by Dr. Muayyad Al-Ubaidi (University of Oklahoma Health Sciences Center, Oklahoma City, OK) [45] and maintained in DMEM/High glucose medium (Gibco, Carlsbad, CA) containing 10% FBS and 1% penicillin/streptomycin at 37°C in a humidified 5% CO2 atmosphere.

#### Co-culture of retinal microglial cells with 661W cells

**Figure 1:**
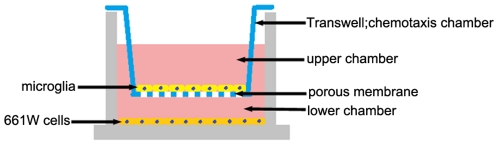
Illustration of the transwell co-culture system and the chemotaxis assay. The transwell consists of two chambers separated by a porous membrane. The 661W cells were placed on the bottom of the lower chamber while the microglial cells were placed on the membrane of the upper chamber.

Freshly collected microglia were reseeded onto 6 well transwell collagen-coated membrane inserts (Corning, Corning, NY). Separately, 661W cells were grown to confluence in a 6 well plate. After 24 hours incubation in their basal medium respectively, the previously prepared transwell inserts containing retinal microglia were then placed into wells with 661W cells. Before light exposure, some inserts of microglia were pretreated by the addition of anti-CX3CR1 neutralizing antibody (Torrey Pines Biolab, La Jolla, CA) or recombinant full-length fractalkine (R&D Systems, Minneapolis, MN) for 5 hours. Then microglia-661W cell co-cultures and controls undergone the light exposure. The 0.4 um pore size of the transwell prevents direct cell–cell interactions but allows the diffusion of soluble factors through the membrane (Fig. 1).

#### Light exposure of the SD rats

SD rats were anesthetized by intraperitoneal injection of ketamine (50 mg/kg) and xylazine (10 mg/kg). Pupils were fully dilated with 1% atropine. The rats were placed separately in cages. After 24 hours' dark adaptation, they were exposed to blue light at an intensity of 2500 lux for 24 hours. After that, they were placed in darkness for another 24 hours and then were returned to the normal light/dark cycle.

#### Light exposure of the 661W cells

The 661W cells were seeded in a 6 or 12 well culture plate (Corning, Corning, NY) for 24 hours followed by 5 hours of intense blue light exposure set at 6000 lux in a light box which was equipped with 16 blue LED lamps fixed on the superior and inferior side. The temperature was maintained at 37°C and the control cells were shielded from light and were kept under conditions similar to those for the cells in the light exposure.

### Histopathology and Immunohistochemistry

Anesthetized rats were transcardially perfused with 0.9% saline, followed by 4% paraformaldehyde (PFA) solution in 0.1 M phosphate buffer. The eyes were enucleated and postfixed in 4% PFA for 2 hours, then transferred to formalin overnight. The eyes were paraffin embedded and sectioned at 5-um thickness. Histopathological evaluation was performed on sections of the ones through optic nerves using hematoxylin and eosin (HE) staining. Three defined regions were selected in the superior hemisphere for measurements: two were located at points 450 µm away from the ora serrata and the optic nerve head, while the third was chosen at the midpoint. Five 100-µm consecutive sections were measured for each region. The thickness of the outer nuclear layer (ONL) was determined by an image-analysis system.

For immunohistochemical studies of retinal sections, after postfixation, the eyecups without anterior segments were transferred to 20%–30% graded sucrose solutions overnight at 4°C and then embedded in OCT compound. Frozen sections were cut at 10 µm thick with a cryostat and the sections were kept in a −20°C freezer until use. The retinal sections or cells were fixed with 4% PFA for 30 min. After being washed with PBS, they were permeabilized with 0.5% Triton for 15 min followed by blocking with 10% goat serum in PBS for 1 hour at room temperature. Subsequently, they were incubated with primary antibodies overnight at 4°C. The following antibodies were used: fractalkine and CX3CR1 antibody (1∶150, Torrey Pines Biolab, La Jolla, CA), OX-42 antibody (1∶200, Abcam, MA, USA), recoverin antibody (1∶200, Santa Cruz Biotechnology, Santa Cruz, CA). After rinsing with PBS, the sections or cells were incubated with appropriate secondary antibodies (Invitrogen, Carlsbad, CA) for 1 hour. Finally they were counterstained with DAPI (Sigma-Aldrich, St. Louis, MO) and examined by laser confocal microscope (Leica Microsystems).

### TUNEL staining

TUNEL was performed using a fluorescent DNA fragmentation detection kit (Roche, Nutley, NJ) according to the manufacturer's instructions. The field was chosen randomly, and nine fields were chosen for every specimen. The number of TUNEL-positive cells was counted to obtain the ratio of TUNEL positivity to the total number of cells.

### Western Blot Analysis

SD rats were sacrificed 2 hours, 6 hours, 1, 3, 5 or 7 days after the light damage. Six retinas were used for each time point. The retinas were homogenized using ultrasound. Equal amounts of proteins were then loaded and separated on SDS-PAGE and were transferred to PVDF membranes (Millipore, Billerica, MA). The membranes were blocked in 5% nonfat milk TBST (20 mM Tris, 150 mM NaCl, 0.1% Tween, pH 7.4) for 30 min and subsequently incubated in primary antibodies overnight at 4°C. After washing, the secondary antibodies were added, and then the labeled proteins were revealed. Quantitative measurement of band intensity was made and normalized against internal controls

### Real-time PCR

The total RNA was extracted using trizol RNA extraction reagent (Invitrogen, Carlsbad, CA), according to the manufacturer's instructions. One microgram of total RNA was reverse-transcribed into cDNA. Real-time PCR was performed using the ABI Prism 7900 Sequence Detection system (Applied Biosystems). The primers for amplifications of fractalkine, CX3CR1, TNF-α,IL-1β were synthesized by Shenggong (Shanghai) company. The reactions were set up with 10 ul SYBR Green PCR Master Mix (Takara, Shuzo, Kyoto, Japan), 1.0 ul 10 umol primer mixture, and 2 ul cDNA template. The real-time PCR was performed following the conditions: 95°C for 10 seconds followed by 35 cycles of indicated temperature and 72°C for 30 seconds. Relative mRNA levels of target genes were normalized to beta-actin.

### ELISA

After light exposure was performed on the cell cultures, the supernatants were collected at 6, 12, 24, 36, 48 hours for enzyme-linked immunosorbent assay (ELISA) measurement. The levels of soluble fractalkine, TNF-α,IL-1β and IL-10 were evaluated with the sandwich ELISA Kit according to manufacturer's instructions (R&D Systems, Minneapolis, MN). Working concentrations of capture and detection antibodies were optimized to increase assay sensitivity. Colorimetric analysis was performed using an Elisa reader. Absorbance was obtained by subtracting readings at 540 nm from readings at 450 nm.

### Chemotaxis assay

The microglial chemotaxis assay was performed with a chemotaxis apparatus that consists of two 12-well chambers separated by a membrane containing 8-µm pores (BD Biosciences, San Jose, CA) (Fig. 1). This assay is based on the premise of providing a gradient of the chemotactic agent and allowing the cells to migrate through a porous membrane toward the chemotactic agent. Briefly, microglia were placed on the membrane of the upper chamber (2.5×10^5^cells/well) while 661W cells were placed on the bottom of the lower chamber (3×10^5^ cells/well). Cells were allowed to adhere to the membranes or plates for 24 hours. To assess the impact of soluble fractalkine on chemotaxis, microglia were pretreated with anti-CX3CR1 antibody for 2 hours in some wells, whereas recombinant soluble fractalkine was added in the lower chamber of some other wells, prior to light exposure. After photic injury for 5 hours, the cells were allowed to migrate through the pores for an additional 1, 7, or 19 hours (6, 12, 24 hours from the beginning of light exposure). Microglial cells are adherent cells which are more likely to attach to the underside of the membrane, so the cells that did not migrate and remained on the upper surface of the membrane were removed with a cotton swab, and the ones that had migrated to the underside of the membrane were stained with DAPI and counted. In at least three independent experiments, three wells per treatment were counted in nine random fields at 50× magnification per well. The number of microglial cells that migrated in response to injured 661W cells was compared to the number of cells that migrated randomly in the control group.

### Statistical Analysis

All results are presented as mean±standard error. Data were analyzed with Student's t tests for comparisons between two groups and with ANOVA for multiple comparisons (GraphPad Prism 5). A value of P<0.05 were considered statistically significant.

## Results

### Light-induced photoreceptor degeneration

**Figure 2:**
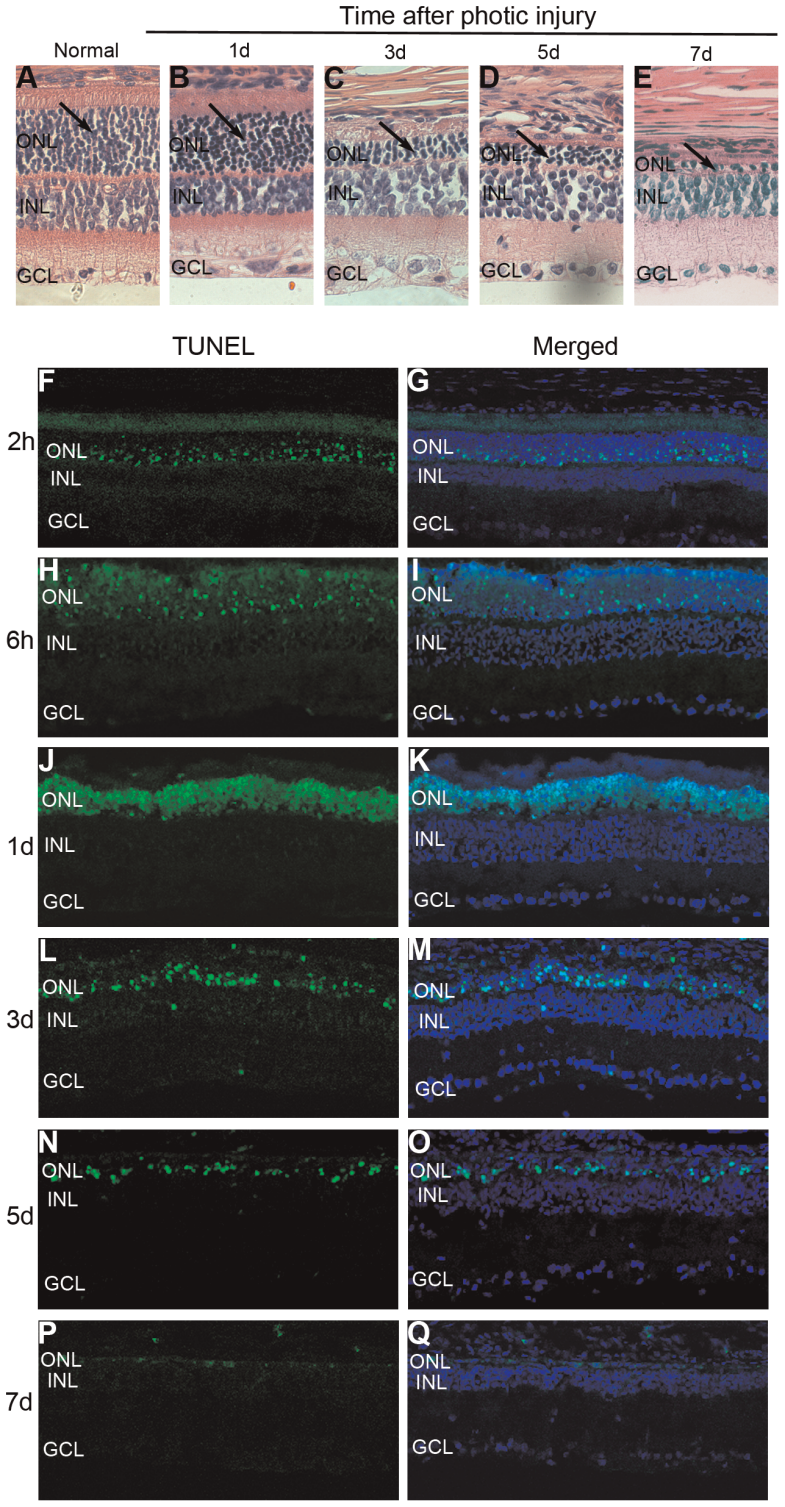
Hematoxylin–eosin and TUNEL staining of SD rat retinas showed progressive changes in the ONL thickness and photoreceptor apoptosis in the in vivo light-induced photoreceptor degeneration model. (A–E) Compared with the normal group, the photoreceptor number and ONL thickness decreased markedly at different time points after exposure to light (black arrow). (F–I) A few hours after exposure to light, TUNEL-positive cells began to appear in the ONL. (J–K) At 1 day, the number of TUNEL-positive cells reached a peak in the ONL. (L–O) TUNEL-positive cells gradually decreased at 3 and 5 days. (P–Q) At 7 days, TUNEL fluorescence was almost completely absent.

**Figure 3:**
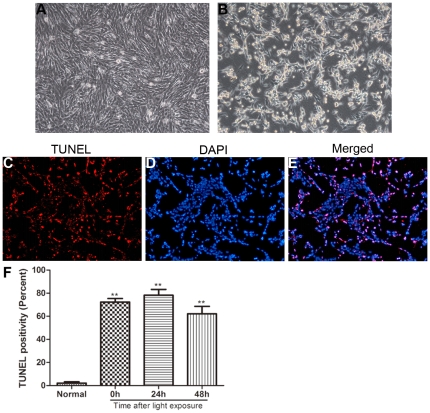
The effect of intense blue light on 661W photoreceptor cell morphology and apoptosis. (A) In the normal control, 661W had a flattened appearance with few intercellular spaces. (B) After light exposure, the cells became spindled with large intercellular spaces. (C–E) 661W photoreceptors underwent apoptosis, as determined by TUNEL assay. (F) Statistical analysis showed that the TUNEL positivity was significantly increased at 0 hour, 24 hours and 48 hours after light exposure.

After 24 hours of blue light exposure, SD rats showed progressive photoreceptor cell loss and a decrease in the ONL thickness, with disorganization of the inner and outer segments of the photoreceptors (Fig. 2A–E). At 7 days after light exposure, the photoreceptors in the posterior retina were almost completely lost, with only one row of cells remaining, and the average ONL thickness was significantly decreased compared with that of normal rats (Fig. 2E). Detection of cell apoptosis during the light-damage process was performed by TUNEL assay. Positive cells were seen in the ONL as early as 2 hours after light exposure and peaked at 1 day (Fig. 2F–K), and then the number of TUNEL-positive cells gradually decreased due to photoreceptor loss (Fig. 2L–Q). In the in vitro experiment, the appearance of 661W cells changed from flat with small intercellular spaces to spindled with large intercellular spaces after exposure to blue light for 5 hours (Fig. 3A–B), and 70% to 80% of them succumbed to apoptosis (Fig. 3C–F).

### Retinal microglial activation and migration in photic injury

**Figure 4:**
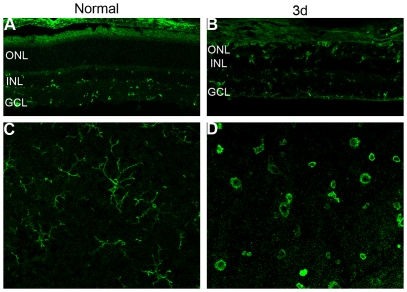
Immunolabeling with the OX42 antibody showed the activation and migration of retinal microglia. (A) In normal retinas, labeled microglia existed only in the GCL and IPL. (B) At 3 days after exposure to light, plenty of microglia migrated to the outer retina. (C–D) The microglial morphology changed from the resting ramified state with long processes to the activated amoeboid appearance at 3 days after photic injury.

In normal retina, OX42-labeled microglial cells were found only in the inner part of the retina, such as in the ganglion cell layer (GCL), the inner plexiform layer (IPL), and the inner nuclear layer (INL) (Fig. 4A). After 24 hours of light exposure, an increasing number of microglia began to migrate into the ONL and the subretinal space (Fig. 4B), appearing to change their morphology from cells with long dendrites to amoeboid cells with retracted processes as they became activated in retinal whole mounts [46] (Fig. 4C–D).

### Expression of fractalkine and CX3CR1 in the retina

**Figure 5:**
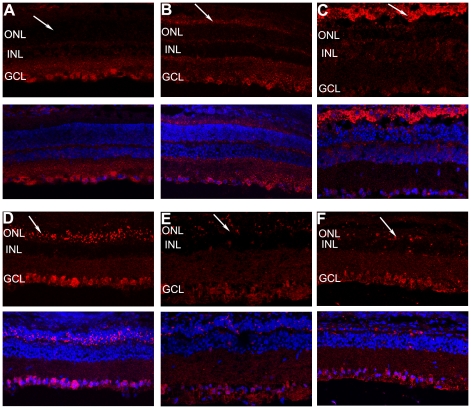
Immunofluorescence analysis of fractalkine in the retina at different time points after exposure to light. (A) In the normal retina, fractalkine immunoreactivity showed a weak but widely distributed fluorescence. (B–F) At 6 hours, 1, 3, 5, 7 days after light exposure, fractalkine staining increased in the photoreceptors.

**Figure 6:**
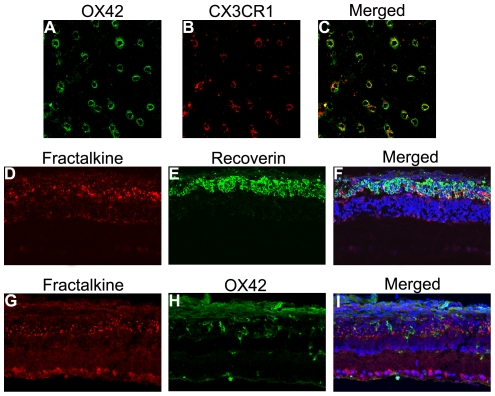
Double immunofluorescence analysis of CX3CR1 and fractalkine at the 3-day time point after light exposure. (A–C) Double staining for OX42 and CX3CR1 revealed that CX3CR1-positive cells were reactive for the microglial marker. (D–I) Photoreceptors were labeled by recoverin antibody and microglial cells were labeled by OX42 antibody. The two merged images showed that photoreceptors were the source of increased fractalkine as well as the direction of microglial migration.

**Figure 7:**
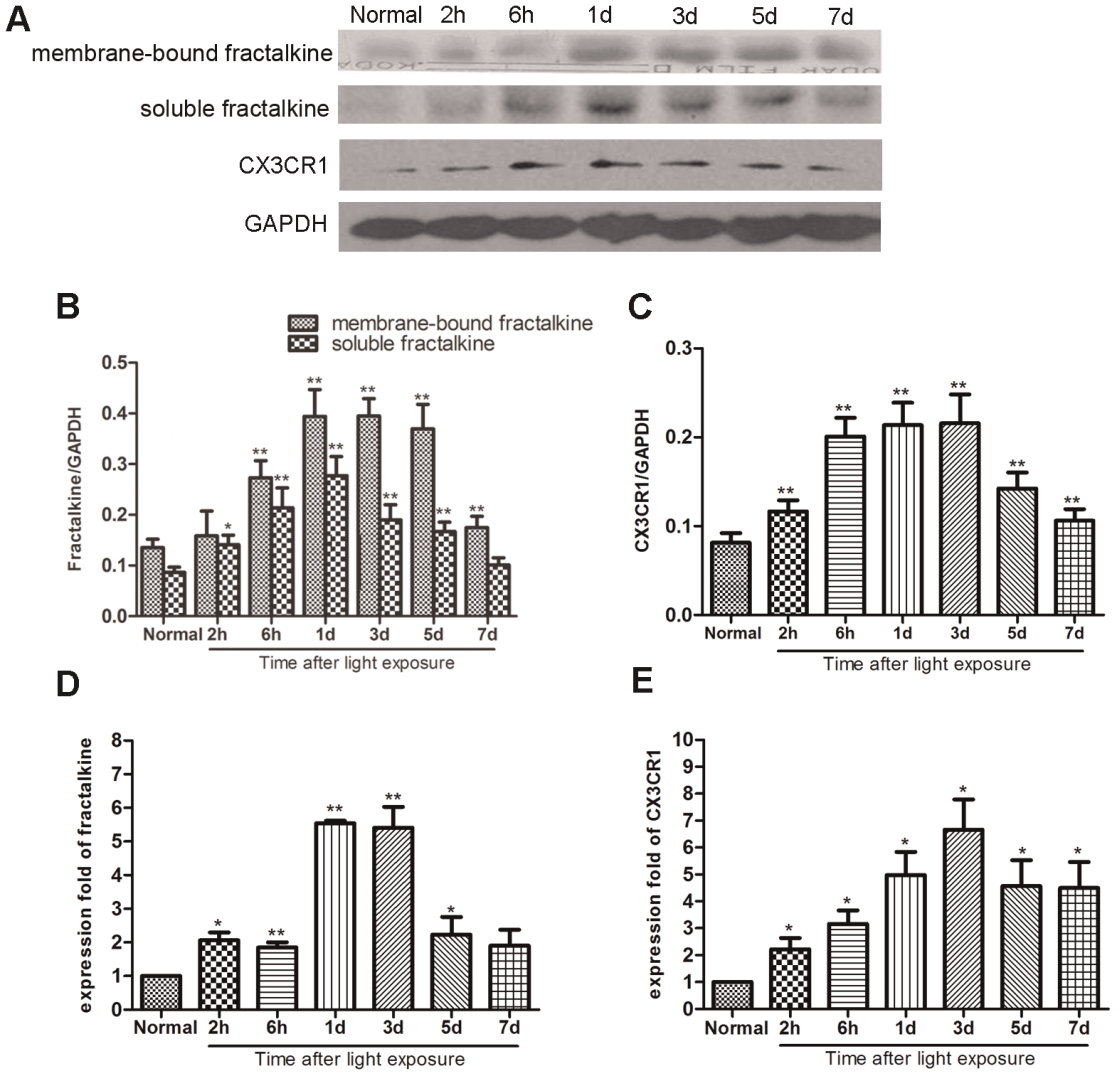
Western blot and real-time PCR analyses for fractalkine/CX3CR1 expression in the retinas after exposure to light. (A–C) The protein expressions of fractalkine and CX3CR1 began to increase at two hours after exposure to light and peaked at 1 day and 3 days respectively, then decreased. (D–E) The mRNA results were normalized relative to levels in the normal control retinas.

**Figure 8:**
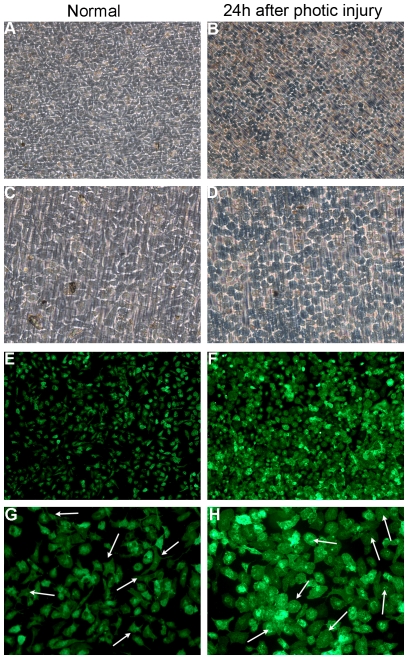
The changes in microglial morphology in the co-cultures after exposure to light. (A, C, E, G) Primary retinal microglia grown in basal media showed ramified shapes with processes. (B, D, F, H) After exposure to light, the microglia in the co-cultures became rounder and took on a characteristic amoeboid shape as they were activated.

**Figure 9:**
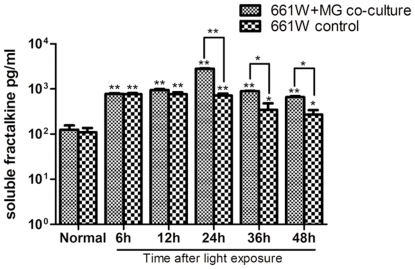
The soluble fractalkine release after exposure to light. ELISA analysis revealed that the levels of soluble fractalkine were increased in the 661W control group and the 661W+MG co-culture group. The difference between them is not statistically significant at 6-hour and 12-hour time points. At other time points, the release of soluble fractalkine in the 661W control group was less than that of the co-culture group due to the further degeneration of remaining photoreceptors caused by activated microglia.

The complementary expression of fractalkine on neurons and CX3CR1 on microglia establishes a unique communication whereby neurons constitutively express and release fractalkine to regulate the function of microglia in various models of central neurodegenerative diseases. This evidence led us to hypothesize that the fractalkine–CX3CR1 axis participates in retinal degeneration. We first confirmed the cellular source of fractalkine and its receptor CX3CR1 in the retina. The level of fractalkine immunoreactivity was low (Fig. 5A), but still clearly detectable throughout the normal control retina, and double-labeled immunofluorescence indicated that CX3CR1-positive cells were exclusively microglia, due to their co-expression of the microglial marker OX-42 (Fig. 6A–C). Next, to investigate whether fractalkine and its receptor CX3CR1 were involved in retinal photic injury, their mRNA and protein expression were examined in the retina at 2 hours, 6 hours, 1, 3, 5, and 7 days after light exposure. The results indicated that CX3CR1 expression was up-regulated (P<0.05) (Fig. 7A, C, E). Similarly, compared with the weak level of expression observed in the normal retina, fractalkine expression, located mainly at the photoreceptor segments and the ONL, was also significantly up-regulated after light damage (Fig. 5B–F, 7A, B, D). At 6 hours and 1 day, its expression began to upregulate at the segment layer first, whereas it appeared mainly at the ONL layer at 3, 5, and 7 days. In photoreceptor–microglia transwell co-cultures, light-damaged 661W photoreceptors resulted in microglial activation and subsequent further degeneration of the remaining photoreceptors (Fig. 8A–H). We then analyzed whether soluble fractalkine was released from light-damaged 661W photoreceptors by evaluating its levels in the cell supernatants. Results from the ELISA assay indicated that the soluble fractalkine was not detected in the single microglial culture group. However, in the co-culture group and the 661W control group, soluble fractalkine was released over time after photic injury, reaching a maximum concentration of about 2.8 ng/ml at 24 hours in the co-culture group (Fig. 9). From this we can conclude that 661W cells were the cellular sources of soluble fractalkine. This evidence also suggested that there is a close spatial and temporal relationship among photoreceptor apoptosis, fractalkine/CX3CR1 impairment, and microglial activation/migration (Fig. 6D–I), indicating that the fractalkine/CX3CR1 ligand-receptor pair might be crucial for photoreceptor-microglial cross-talk.

### Up-regulated IL-1β and TNF-α expression after photic injury

**Figure 10:**
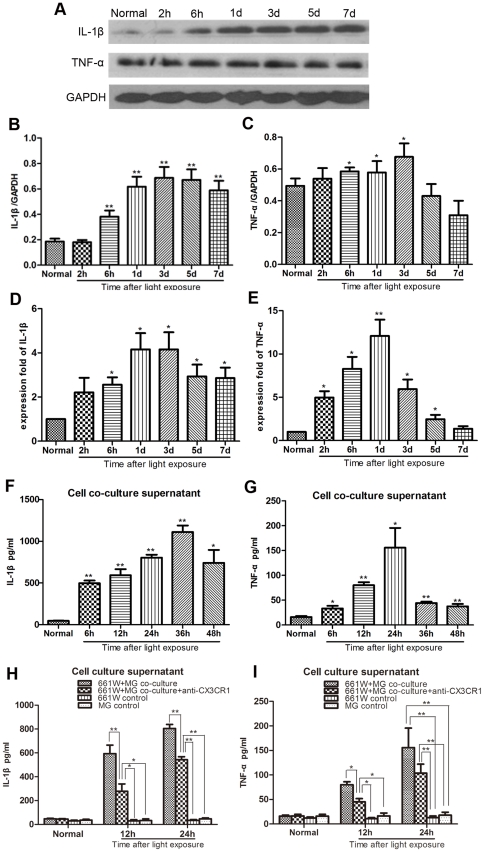
The expression of IL-1β and TNF-α after exposure to light. (A–C) The protein expressions of IL-1β and TNF-α in the retina began to increase at 6 hours after exposure to light and peaked at 3 days. (D–E) The mRNA expression of IL-1β and TNF-α in the retina was increased, and peaked at 1 day after photic injury as detected by real-time PCR. (F–G) ELISA assay detected the upregulated secretion of IL-1β and TNF-α in the 661W/microglia co-culture supernatant. (H–I) Compared with untreated co-cultures, the group pretreated with neutralizing CX3CR1 antibody showed significantly decreased expression of IL-1β at 12 hours and 24 hours after exposure to light. For TNF-α, statistical analysis showed significantly reduced production in the treated cultures at 12 hours. No difference was found between treated and untreated co-cultures at 24 hours.

Activated microglia are known to produce pro-inflammatory cytokines such as IL-1β and TNF-α that result in local inflammatory responses and neurodegeneration [47], [48]. Therefore, we examined the levels of IL-1β and TNF-α at different time points after exposure to light. Compared with the low levels in the control retinas, the expressions of both IL-1β and TNF-α were up-regulated in a time-dependent manner (Fig. 10A–E). ELISA analysis also revealed that the levels of these factors were increased in the co-culture cell supernatants (Fig. 10F–G).

### Inhibition of light-induced IL-1β and TNF-α release by the anti-CX3CR1 antibody

**Figure 11:**
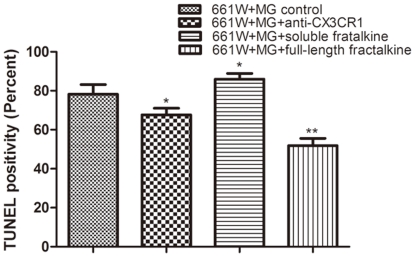
Statistical analysis of TUNEL assay in different co-culture groups at 24 hours after exposure to light.

The increased release of soluble fractalkine after light exposure prompted us to investigate whether it plays an active role in microglial activation combined with enhanced pro-inflammatory properties. Microglia were pretreated with a neutralizing antibody against CX3CR1 for 6 hours, followed by measurement of the expression of inflammatory cytokines. As a result, the partial blockade of CX3CR1 function markedly reduced the production of IL-1β and TNF-α (Fig. 10H–I). In addition, morphometric analysis showed that the CX3CR1 antibody treated co-cultures had significantly higher numbers of photoreceptors remaining than did the untreated ones (data not shown). Also the result of TUNEL assay indicated that the CX3CR1 antibody treated co-cultures had lower percentage of TUNEL-positive photoreceptors than did the untreated ones at 24 hours after exposure. (Fig. 11)

### Soluble fractalkine-induced migration of microglia

**Figure 12:**
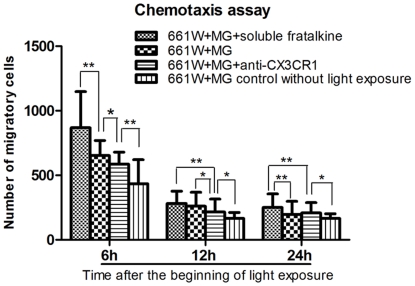
Soluble fractalkine-induced microglial migration in the co-culture of retinal microglial cells with 661W cells. Results are presented as numbers of migratory cells. Microglia exhibited stronger migratory activity in the co-cultures with additional soluble fractalkine than in other groups and there was a significant reduction of microglial migration in the co-cultures with neutralizing CX3CR1 antibody at most time points. The most obvious result was obtained at the 6-hour time point after the beginning of light exposure.

Chemotaxis assays were performed in the microglia-photoreceptor co-cultures. Our data show that microglia exhibited stronger migratory activity in the cultures with additional recombinant soluble fractalkine, suggesting that soluble fractalkine released by photoreceptors after photic injury contributes to the migratory behavior of microglia in vitro (Fig. 12). When microglia were pretreated with neutralizing antibody against CX3CR1 for 2 hours, the effect of soluble fractalkine on migration was partly attenuated (Fig. 12). These findings suggest that the fractalkine/CX3CR1 axis is involved in microglia migration. In addition, TUNEL assay showed that the soluble fractalkine treated co-cultures had higher percentage of TUNEL-positive photoreceptors than did the untreated ones at 24 hours after exposure (Fig. 11). This might be due to the reason that the increased level of soluble fractalkine leads to increased recruitment of activated microglia and excess production of inflammatory cytokines, which will cause inflammation-mediated neurodegeneration of the remaining photoreceptors.

### Neuroprotective effect of membrane-bound fractalkine against light-induced toxicity in microglia/photoreceptor co-cultures

**Figure 13:**
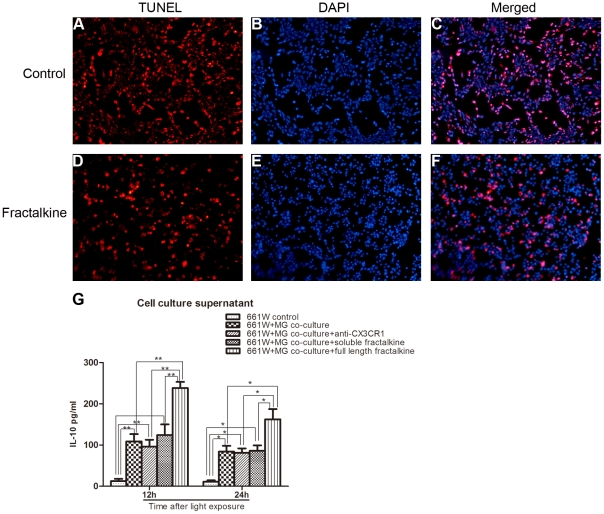
The effect of membrane-bound fractalkine on 661W photoreceptor survival. (A–C) At 24 hours after exposure to light, a large number of TUNEL-positive photoreceptors was detected in the untreated co-culture. (D–F) TUNEL-positive cells were less abundant in the co-culture treated with exogenous membrane-bound fractalkine. (G) The expression of anti-inflammatory cytokine, IL-10, was increased in the recombinant full-length fractalkine treated groups.

To test whether membrane-bound fractalkine upregulation has any role in neuroprotection after photic injury, as suggested by previous studies [49], [22], we applied recombinant full-length fractalkine to the upper chamber. Light-induced cell death was attenuated in the cultures pretreated with exogenous fractalkine compared with controls. This protection was further confirmed by TUNEL assay at 24 hours after exposure (Fig. 13A–F). Statistical analysis showed that the percentage of TUNEL-positive 661W cells was significantly lower in the fractalkine treated (51.9±3.7%) than in the control group (78.2±5%, P<0.05) (Fig. 11). Moreover, the expression of IL-10, one of the anti-inflammatory cytokines [50], was increased in the full-length fractalkine treated co-cultures, as measured by ELISA assay (Fig. 13G).

## Discussion

In the present study, we sought to determine whether fractalkine and its sole receptor CX3CR1 were involved in photoreceptor-microglia cross-talk after blue photic injury, resulting in microglial activation as well as migration, and subsequently contributing to the further apoptosis of remaining photoreceptors. First we confirmed the constitutive expression of fractalkine/CX3CR1 by photoreceptors and microglia respectively in the retina under normal and light-damaged conditions. Moreover, the extent of photoreceptor degeneration and microglial migration paralleled the increased level of fractalkine/CX3CR1. In addition, we noted that soluble fractalkine seemed to play a certain role in enhancing the release of inflammatory cytokines and facilitating microglial migration. However, recombinant full-length fractalkine might exert anti-inflammatory effects via IL-10 release and potential control of microglial activation.

Microglia are the resident immune cells in the CNS that perform dynamic immune surveillance [51]. In the physiological state, microglia exist in a resting appearance characterized by a ramified morphology with protruding processes surveying and monitoring their microenvironment [52], [53]. In response to pathological stimuli, microglia undergo rapid activation and quickly migrate to the damaged site to perform the function of macrophages, including providing innate immunity, phagocytosis, cellular maintenance, and neurotrophic secretions [54], [55]. However, microglia can also promote inflammation and act as contributors to progressive neuron damage in several neurodegenerative diseases [56]. Substantive evidence indicates that microglial cells in the retina play an important role in the development of retinal degenerative diseases [57]. The current research confirmed that light-induced photoreceptor apoptosis is often accompanied by microglial activation and migration from the inner retinal layer to the outer retina and even the subretinal space, combined with the upregulated expression of pro-inflammatory factors including IL-1β and TNF-α [58]. Furthermore, we showed a neuroprotective effect of naloxone against light-induced photoreceptor degeneration through inhibition of retinal microglial activation [9]. These observations suggest that injured photoreceptors must release certain signaling molecules that attract and activate microglia, resulting in further degeneration of remaining photoreceptors. Although the interaction between neurons and microglia are likely critical in regulating their function and survival, knowledge of the relevant signaling cytokines needs to be further clarified.

Fractalkine is a relatively new member of the chemokine family that is reported to be constitutively expressed by neurons, whereas its sole receptor, CX3CR1, is mainly expressed by microglia in the CNS [59]. Different from many other chemokines, fractalkine is synthesized as a transmembrane protein, which can be cleaved into a soluble form in certain pathological conditions [60]. This specific expression pattern implies a role in mediating neuron-microglia cross-talk. In the present research, we first established a rat photic injury model to investigate whether there was a change in fractalkine/CX3CR1 expression in the retina, and whether the migration of microglia to the injured neurons was associated with fractalkine/CX3CR1 expression. Western blot and real-time PCR analysis revealed that the levels of fractalkine/CX3CR1 were elevated after light exposure, with the increased expression site of fractalkine exclusively in the photoreceptors. Then, in an in vitro cell co-culture study, increased soluble fractalkine accumulation was detected by ELISA assay. This means that part of the membrane-bound form fractalkine was cleaved and released as soluble protein. We also noted that the apoptotic peak of photoreceptors at 1 day was consistent with that of the elevated level of fractalkine, and both were ahead of the peak of microglial infiltration at 3 days. Taken together, these findings suggest the possibility that, after exposure to intense blue light, soluble fractalkine is initially released by injured photoreceptors, and thereby causes the migration of microglia into the ONL via CX3CR1. Moreover, these microglia produce toxic agents which in turn exacerbate the progressive neurotoxic consequences.

Interestingly, it seems to be organ- and disease-specific for fractalkine/CX3CR1 to exert either a toxic or a protective effect [61]–[62]. On one hand, inactivation of fractalkine or CX3CR1 reduces the severity of some diseases [24], [63]. On the other hand, fractalkine/CX3CR1 signaling represents a potential protective effect on neurons in many degenerative CNS diseases [22], [61]. For instance, CX3CR1 deficiency dysregulates microglial responses, resulting in neurotoxicity in models of Parkinson's disease and amyotrophic lateral sclerosis [54]. Moreover, adding extra fractalkine protects hippocampal neurons from glutamate- and HIV-1-induced neurotoxicity [49], [22].

In ocular tissues, reports on the role of fractalkine/CX3CR1 signaling are also controversial. In one study, CX3CR1 deficiency showed reduced macrophage accumulation but severe neovascularization after alkali injury in the cornea [64]. However, in some other studies, the same CX3CR1 deficiency conversely led to microglia accumulation in the ONL and subsequent neuronal degeneration and neovascularization in an AMD model [65], as well as in experimental autoimmune uveitis, resulting in a significant increase in neuron apoptosis and prolonged inflammatory response [66]. In this regard, it was our interest to determine whether fractalkine/CX3CR1 signaling has potential neurotoxic or neuroprotective effects on light-induced photoreceptor degeneration. In the photoreceptor-microglial transwell co-cultures, we showed that blockade of fractalkine/CX3CR1 interaction by neutralizing antibody against CX3CR1 ameliorated the microglial inflammatory response to damaged photoreceptors and improved the photoreceptor survival rate. In addition, microglial migration was found to be attenuated by this antibody. Our data also showed a significant increase in microglial migration and production of TNF-α and IL-1β in vitro in response to exogenous recombinant soluble fractalkine adding, suggesting that soluble fractalkine cleavage and distribution in the retina could affect activation and migration of microglia in vivo. In contrast, the pretreatment of exogenous full-length fractalkine likely exerted neuroprotective action on 661W photoreceptors. This effect might be mediated by promotion of microglial anti-inflammatory cytokine IL-10 release or by other mechanisms that remain to be studied.

Taken together, these results strongly suggest that fractalkine/CX3CR1 signaling exerts multiple effects on the cross-talk between microglia and photoreceptors. The soluble form of fractalkine released from photoreceptors may function as a chemotactic factor to cause the activation and migration of retinal microglia, while fractalkine in low concentrations in the membrane-bound form may exist as a regulator of the beneficial balance between microglia and photoreceptors. The reasons for these differences are not quite clear, but it has been hypothesized that CX3CR1 activates different signaling pathways in different situations [65]. However, these intricate effects of fractalkine/CX3CR1 in the co-cultures reported herein need extensive investigation in animal models.

In conclusion, changes in the expression of fractalkine/CX3CR1 in response to intense blue light injury that we have documented strongly suggest that this ligand–receptor signaling pathway may play a role in the process of retinal microglial activation and migration in light-induced photoreceptor degeneration. Our results indicate that blockage of soluble fractalkine represents a decreased inflammatory response, whereas overexpression of full-length fractalkine suggests a role in photoreceptor survival. In this regard, we will further explore potential downstream intracellular signal transduction pathways in the future work.
